# Management of temporomandibular joint arthritis in adult rheumatology practices: a survey of adult rheumatologists

**DOI:** 10.1186/1546-0096-10-26

**Published:** 2012-08-20

**Authors:** Sarah Ringold, Nikolay Tzaribachev, Randy Q Cron

**Affiliations:** 1 Pediatrics, Seattle Children’s Hospital, 4800 Sandpoint Way NE, Seattle, WA, 98105, USA; 2 Center for Rheumatic Disease, Bad Bramstedt, Germany; 3 Pediatric Rheumatology, University of Alabama-Birmingham, 1530 Third Ave South, SHEL 176, Birmingham, AL, 35294-2182, USA

**Keywords:** Temporomandibular joint arthritis, Juvenile idiopathic arthritis, Rheumatoid arthritis

## Abstract

**Background:**

The temporomandibular (TMJ) is frequently involved in juvenile idiopathic arthritis (JIA), however little is known about management of this joint once a patient transitions from pediatric to adult care and about how rheumatologists approach TMJ involvement in rheumatoid arthritis (RA). The objective of this project was to describe adult rheumatologists’ approaches to the diagnosis and treatment of TMJ arthritis in adults with JIA or RA.

**Findings:**

One hundred and eighteen rheumatologists responded to an online survey of adult rheumatologists in the United States and Canada. Respondents estimated that 1-25% of their patients with RA or JIA had TMJ arthritis. Respondents reported lower rates of MRI use (19%) and higher rates of use of splinting/functional devices (50%) than anticipated. Approximately 80% of respondents reported that their practice had a standardized approach to the evaluation of patients with TMJ arthritis. The most commonly used medical therapies were non-steroid anti-inflammatory drugs, anti-tumor necrosis factor alpha medications, and methotrexate.

**Conclusions:**

Despite the majority of respondents stating that their practices had a standardized approach to the diagnosis and treatment of TMJ disease, there nevertheless appeared to be a range of practices reported. Standardizing the evaluation and treatment of TMJ arthritis across practices may benefit both adult and pediatric patients.

## Findings

The temporomandibular joint is frequently involved in juvenile idiopathic arthritis (JIA) with a prevalence as high as 75% [1,2]. A significant proportion of children with JIA and TMJ involvement have been reported to have radiographic progression of their TMJ damage over time [3]. Longitudinal follow-up of children with JIA and TMJ involvement has also indicated that children with TMJ arthritis are more likely to report symptoms of TMJ dysfunction, including headache, neck pain and difficulty with mouth opening, in adulthood than healthy controls [4]. However few data are available regarding the evaluation and treatment of these patients with JIA once they transition from receiving their care from a pediatric rheumatologist to an adult rheumatologist. The objectives of this report were to assess how adult rheumatologists evaluate and treat adult patients with JIA and known or suspected TMJ involvement, and to compare these data to their practice for their patients with rheumatoid arthritis (RA).

## Patients and methods

A 20-question survey was developed using SurveyMonkey^TM^. Participant were asked to answer a set of questions about their care of adult patients with JIA (patients with arthritis diagnosed < 16 years of age) and a separate set of questions about their patients with RA. The survey questions were beta-tested among a group of pediatric rheumatologists prior to distribution. The link to the survey was subsequently distributed via electronic mail to members of the Alabama Society for the Rheumatic Diseases Listserv, to Canadian American College of Rheumatology (ACR) members, to the state specific ACR Listservs, and to a random selection of ACR members within the United States.

Descriptive statistics were generated using the statistical analysis software embedded within the SurveyMonkey^TM^ site.

Approval for this study was obtained from the Seattle Children’s Hospital institutional review board.

## Results

One hundred and eighteen responses were received. The response rate for this survey could not be calculated as we were not able to assess the number of email addresses which were active or the number of active members within each Listserv, and unable to assess the number of surveys actually received (e.g. versus those routed to spam folders). The majority of respondents (86.5%) practiced within the US and had been in practice >15 years (67.8%) since completion of fellowship training. Approximately half of respondents provided care for both children and adults in their practice. Ninety-three percent of respondents cared for at least one adult patient with JIA in their practice.

The majority of physicians (58%) estimated that between 1-25% of their adult patients with JIA had a history of TMJ arthritis and approximately 60% of their adult patients with JIA were currently being treated for active TMJ disease. Similarly, respondents estimated that between 1-25% of their patients with RA had TMJ arthritis and were being actively treated for it.

**Figure 1:**
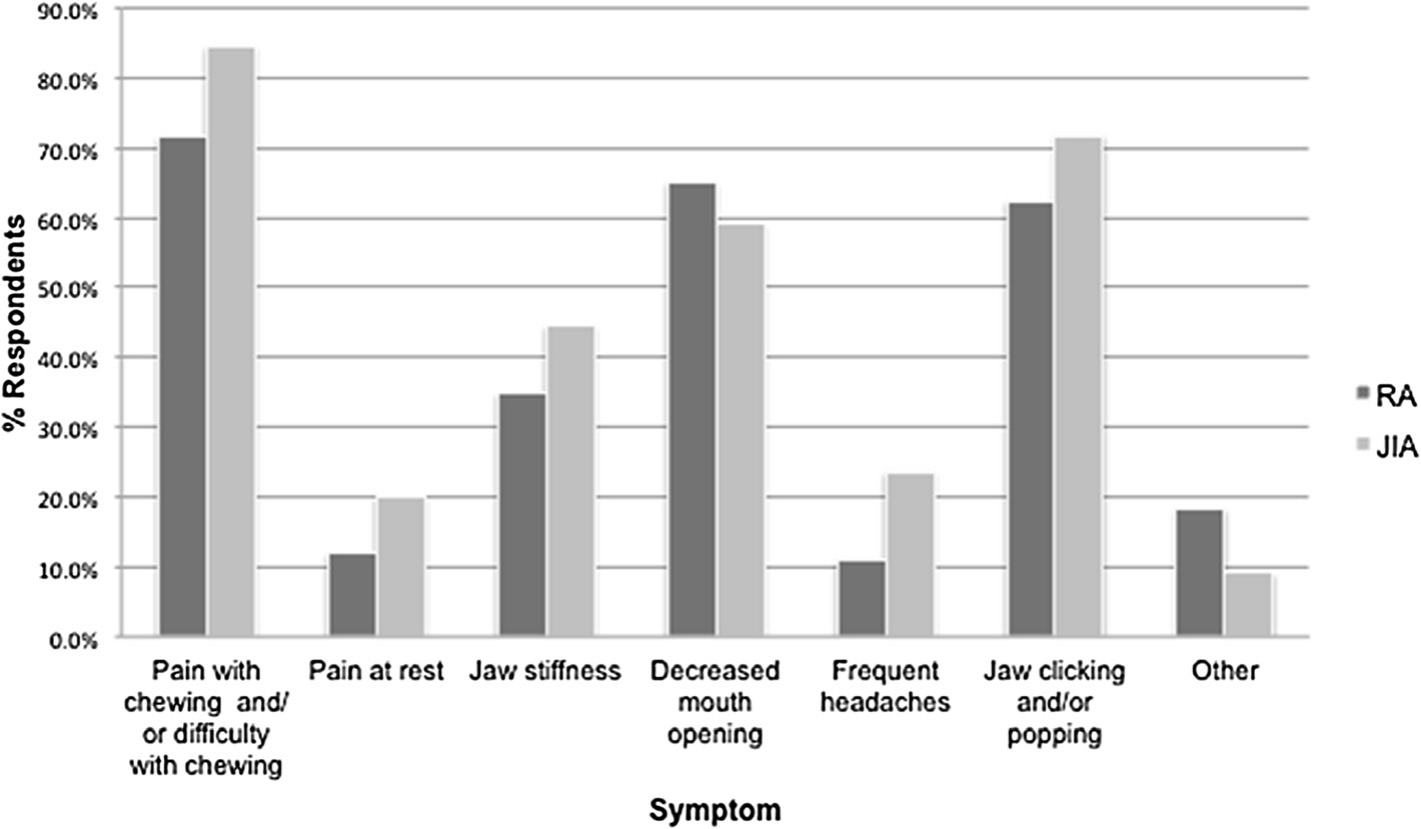
**Tempromandibular joint symptoms.** RA: Rheumatoid Arthritis; JIA: Juvenile idiopathic arthritis.

The majority of physicians estimated that between 1-25% of their adult patients with JIA currently had symptoms of TMJ arthritis. This was similar to their estimates for their patients with RA, although several respondents also noted that they found it difficult to distinguish TMJ arthritis symptoms from TMJ dysfunction in their patients with RA. The most common symptoms were pain with chewing and/or difficulty chewing, decreased mouth opening, and jaw clicking or popping (Figure 1).

**Figure 2:**
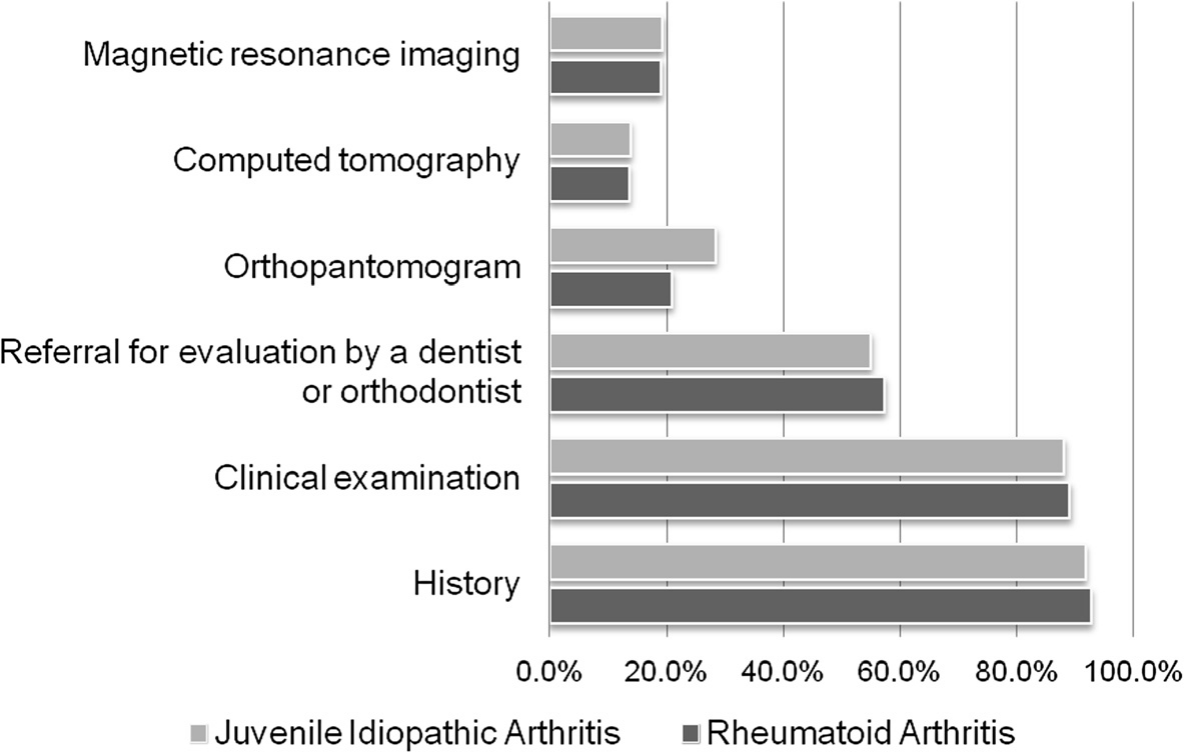
Evaluation of temporomandibular joint arthritis.

Physicians most frequently reported evaluating TMJ arthritis in their adult patients with JIA and in their patients with RA by history and physical, and by referral to dentist or orthodontist (Figure 2). Orthopantomogram was the most frequently used imaging modality, used by 28% of providers for patients with RA and by 20% for patients with JIA. Magnetic resonance imaging was used by 19% of providers for assessing TMJ arthritis in both sets of patients. Approximately 75-80% of respondents reported that their practice did have a standardized approach to the evaluation of TMJ arthritis in patients with either diagnosis.

**Figure 3:**
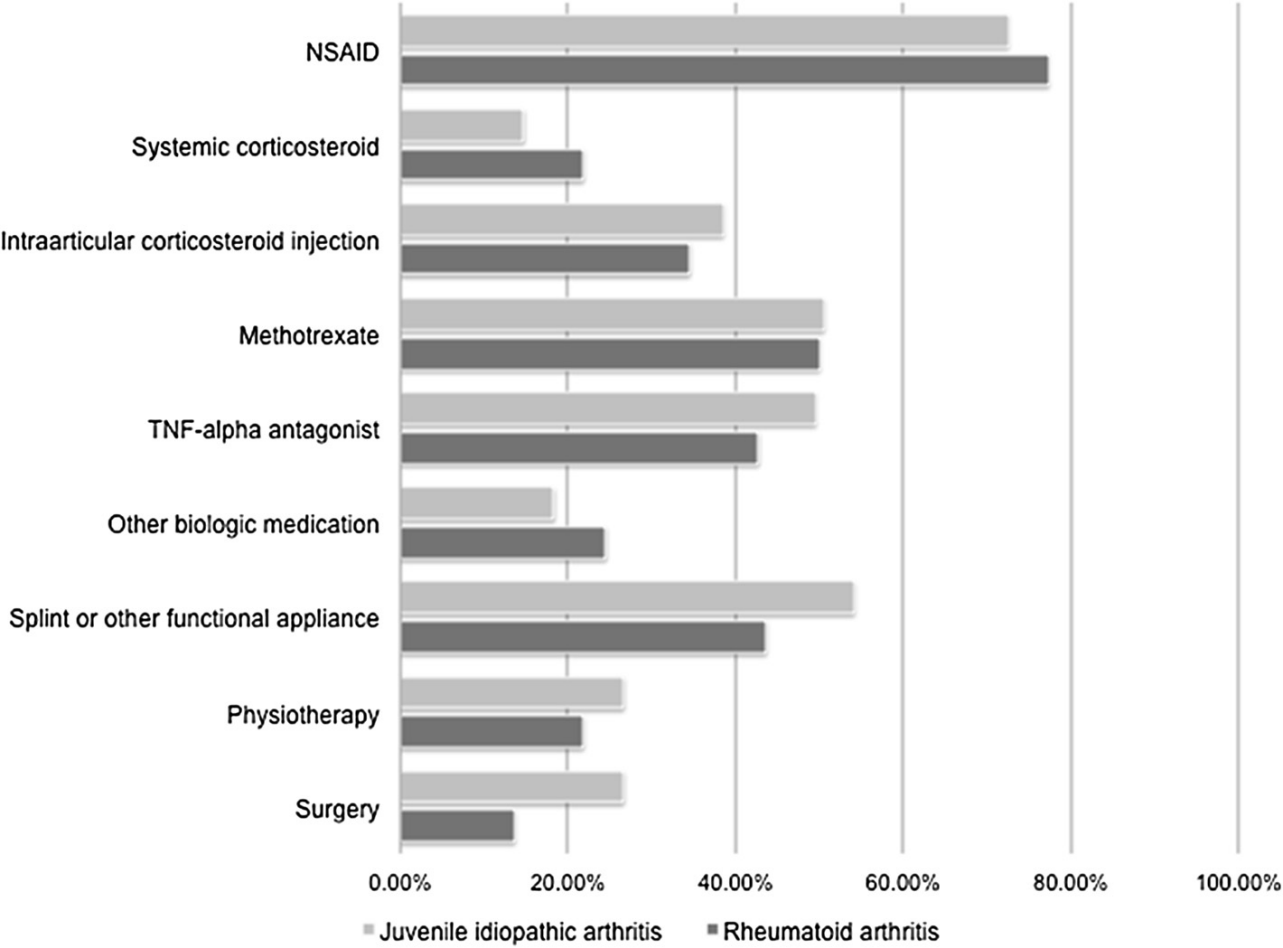
**Treatment of temporomandibular joint arthritis.** TNF-alpha: Tumor necrosis factor-alpha; NSAID: Non-steroidal anti-inflammatory drug.

The systemic medical therapies most commonly used specifically for the treatment of TMJ arthritis included non-steroidal anti-inflammatory drugs, methotrexate, and tumor necrosis factor-alpha inhibitors (Figure 3). Over half of respondents reported using functional orthodontic devices (e.g. soft splints and activator splints) as therapy, and over one-third reported using intra-articular corticosteroid injections.

## Discussion

The above results suggest that adult rheumatologists approach the evaluation and treatment of TMJ arthritis in adults with JIA and those with RA similarly. However, these results highlight two important differences in practice between pediatric and adult rheumatologists. Although the high prevalence of TMJ arthritis in JIA is now well-recognized by pediatric providers, respondents to this survey estimated that only as many as 25% of their adult patients with JIA had TMJ involvement. Similarly, while published series indicate that the prevalence of TMJ involvement in RA may be as high as 45%, the majority of respondents estimated that only as many as 25% of their patients with RA had TMJ involvement [5]. Because we would anticipate that the rheumatologists who responded to this survey might have a particular interest in the TMJ, it is likely that awareness of the high prevalence of TMJ involvement in adults with JIA would be even lower among the broader community of adult rheumatologists. This discrepancy suggests that improved communication between pediatric and adult providers regarding the high prevalence of TMJ involvement in JIA is still needed. Secondly, only a small percentage of respondents indicated using MRI for imaging of the TMJ. MRI with gadolinium is considered the most sensitive modality for detecting active TMJ arthritis in children [1]. While orthopantomogram may define condylar damage, it does not provide information about whether there is synovitis that would impact treatment decisions. In terms of therapy, almost half of the adult rheumatologists reported using systemic methotrexate and/or TNF inhibitors. And while these therapies may help, TMJ arthritis can still occur while taking these systemic treatments [6-8]. Interestingly, almost 40% of adult rheumatologists reported using intra-articular corticosteroid treatments, as has been championed in pediatric rheumatology [9]. Lastly, the number of respondents who reported using splints and/or functional orthodontic appliances to treat TMJ arthritis in adults with JIA or RA was higher than anticipated. While the anticipated effects of splinting are different for adults versus children, the recent data regarding the potential benefit of splinting on dentoalveolar development in JIA, with subsequent improvements in mandibular growth, suggest that splinting may be a treatment modality that pediatric rheumatologists will begin to use more frequently as well [10,11].

Although this survey was limited by its small sample size and reliance on participants’ recall, and may not be representative of the larger community of adult rheumatologists, the results nevertheless highlight the value of the development of standardized practice around TMJ arthritis and the specific discussion of TMJ arthritis, when relevant, during the transition of patients between pediatric and adult providers.

## Competing interest

There are no competing of interest for the authors above and this work.

## Authors’ contributions

SR, NT, RC contributed to the design of the survey. SR and RC participated in administration of the survey. SR conceived of the study and drafted the manuscript. NT and RC read, contributed to, and approved the final manuscript. All authors read and approved the final manuscript.
